# Absorption and Metabolism of the Natural Sweeteners Erythritol and Xylitol in Humans: A Dose-Ranging Study

**DOI:** 10.3390/ijms23179867

**Published:** 2022-08-30

**Authors:** Valentine Bordier, Fabienne Teysseire, Frank Senner, Götz Schlotterbeck, Jürgen Drewe, Christoph Beglinger, Bettina K. Wölnerhanssen, Anne Christin Meyer-Gerspach

**Affiliations:** 1 St. Clara Research Ltd., St. Claraspital, 4002 Basel, Switzerland; 2 Faculty of Medicine, University of Basel, 4001 Basel, Switzerland; 3 Institute for Chemistry and Bioanalytics, School of Life Science, FHNW University of Applied Sciences and Arts Northwestern Switzerland, 4132 Muttenz, Switzerland; 4 Department of Clinical Pharmacology and Toxicology, University Hospital Basel, 4001 Basel, Switzerland

**Keywords:** erythritol, xylitol, erythronate, natural sweeteners, absorption, metabolism, obesity, diabetes

## Abstract

The natural sweeteners erythritol and xylitol might be helpful to reduce sugar consumption and therefore prevent obesity and diabetes. The aim of the present study was to determine the absorption and metabolization into erythronate of different concentrations of erythritol and xylitol. Seventeen healthy lean participants received intragastric solutions of 10, 25, or 50 g erythritol or 7, 17, or 35 g xylitol on three study days in a randomized order. The study was double blinded with respect to the doses administered. We assessed plasma concentrations of erythritol, xylitol, and erythronate at fixed time intervals after administration with gas chromatography-mass spectrometry. We found: (i) a dose-dependent and saturable absorption of erythritol, (ii) a very low absorption of xylitol, (iii) a dose-dependent metabolization of erythritol into erythronate, and (iv) no metabolization of xylitol into erythronate. The implications of the metabolization of erythritol into erythronate for human health remain to be determined and more research in this area is needed.

## 1. Introduction

The still-steady rise in sugar consumption is a key contributor to the dramatic global rise in obesity and associated metabolic disorders, especially type 2 diabetes mellitus. The WHO has proposed a reduction in sugar intake as a preventive and therapeutic strategy to curb these disorders [1]. A possible solution to achieve a reduction in sugar intake is the partial substitution of table sugar and added sugars with low-caloric, naturally occurring bulk sweeteners, also called polyols. Polyols are mono- and polysaccharides in which a carbonyl group is replaced by an alcohol (hydroxyl) group. Polysaccharide polyols are difficult to digest and metabolize due to their hydroxyl groups and their glycosidic linkages other than α1-4 and α1-6 [2]. Monosaccharide polyols, such as erythritol and xylitol, are partly absorbed by passive diffusion along a concentration gradient in the small intestine [3]. These monosaccharide polyols are gaining popularity among patients with overweight and diabetes thanks to their low glycemic indexes [2], which gives them anti-hyperglycemic and anti-diabetic properties [4]. In addition, erythritol and xylitol induce the secretion of gastrointestinal satiation hormones (such as cholecystokinin (CCK) and glucagon-like peptide-1 (GLP-1)) and promote satiety and slow gastric emptying [5,6,7]. Furthermore, the two polyols actively benefit oral health [5].

**Figure 1:**
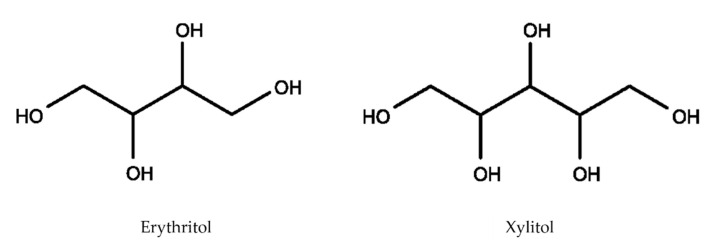
Chemical structures of erythritol and xylitol.

Erythritol is a four-carbon sugar alcohol (C_4_H_10_O_4_, see Figure 1) with a molar mass of 122.12 g/mol and a glycemic index of 0 (in comparison, sucrose and glucose have glycemic indexes of 65 and 100, respectively). In 1996, Bornet et al. [8] found that plasma and urine levels increased within two hours proportionally to the amount of erythritol ingested. They found that the total urinary excretion reached 78% of ingested erythritol after 24 h [9]. A chronic intake of erythritol over seven days showed that 78% of ingested erythritol was excreted in the urine [10]. Munro et al. [11] summarized that erythritol is rapidly absorbed up to 90% by the gastrointestinal tract and quantitatively excreted unchanged with the urine. Whether the remaining 10% of the erythritol dose is fermented in the colon or excreted unchanged via the stool is unknown in humans. However, in an in vitro setting, erythritol was shown to be completely resistant to bacterial fermentation within 24 h [12]. In conclusion, available data suggest that erythritol is mainly absorbed in the intestine, not metabolized by the body, and excreted unchanged via the kidney. However, in a side experiment of their study on metabolic markers of adiposity gain, Hootman et al. [13] recently shed light on an unknown pathway of erythritol metabolism. In their study, three healthy males ingested a single dose of 50 g erythritol and gave finger-prick blood samples at regular intervals after ingestion. The authors observed an immediate increase in blood erythritol concentrations, followed by an increase in erythronate concentrations. They suggest that ingested erythritol is oxidized into the sugar erythrose (C_4_H_8_O_4_), which is in turn oxidized to erythronate (C_4_H_7_O_5_^−^). The authors suggest that 5–10% of the ingested amount of erythritol is metabolized into erythronate [13]. These results lead to new questions about the metabolization of erythritol and the role of erythritol and its metabolites, especially erythronate, for human health in relation to obesity and diabetes.

Xylitol is a five-carbon sugar alcohol (C_5_H_12_O_5_, see Figure 1) with a molar mass of 152.15 g/mol and a glycemic index of 13. Due to its higher molar mass, xylitol is absorbed in smaller proportions than erythritol. There are only a few studies investigating the absorption and metabolism of xylitol. In 1973, Asano et al. [14] studied the intestinal absorption of oral xylitol by aspiration and analysis of ileal content in five healthy subjects. They found that xylitol absorption ranged from 49–95%. However, they did not find any xylitol in plasma samples one and two hours after ingestion, nor did they notice significant amounts in urine up to 24 h after ingestion. After being absorbed, monosaccharide polyols can be excreted unchanged via the kidneys, oxidized directly, or metabolized in the liver to glycogen or glucose [15]. The latter is what Asano et al. [14] suggested as a hypothesis for the absence of xylitol in the blood, while at least half of the ingested quantity was absorbed. Other estimates of xylitol intestinal absorption range from 48% [15] over 53% [16] up to 75% [17]. Xylitol is fermentable by colonic microorganisms and is considered a prebiotic, as it promotes the proliferation and metabolic activity of beneficial bacteria and the production of short-chain fatty acids such as butyrate [18,19]. Livesey summarized the evidence and suggested a consensus of around 49% for absorptive capacity, less than 2% for urinary excretion, and approximately 50% for fermentation [2]. However, like for erythritol, the metabolism of absorbed xylitol still needs further investigation to understand its effect on the human body better and evaluate its potential as a sugar substitute for patients with obesity and diabetes.

We administered different doses of each substance to healthy volunteers to investigate the enteral absorption of erythritol and xylitol and their potential metabolization into erythronate. We showed that the absorption of erythritol and its metabolization into erythronate occur in a dose-dependent manner. The absorption of xylitol was low, and no metabolization into erythronate took place.

## 2. Results

All participants tolerated the study treatments well, and there were no adverse events that led to study discontinuation. Therefore, complete data from 2 × 12 participants were available for analysis. There was no abdominal pain, nausea, flatulence, or vomiting reported after any dose of erythritol or xylitol. One participant had diarrhea after 10 g erythritol. Four participants reported feelings of bloating (after 25 g and 50 g erythritol and after 17 g and 35 g xylitol) and two participants reported increased eructation (after 10 g erythritol and after 7 g xylitol). A subjective increase in bowel sounds was reported by nine participants after 10 g erythritol, seven after 25 g erythritol, and eight after 50 g erythritol; and nine participants after 7 g xylitol, eight after 17 g xylitol, and nine after 35 g xylitol.

### 2.1. Absorption of Erythritol and Xylitol

The absorption of erythritol occurred in a dose-dependent manner. The area under the curve from 0 to 180 min (AUC_180_) and the maximum erythritol plasma concentrations (C_max_) increased in response to the three intragastric loads (AUC_180_: 10 g vs. 25 g, *p* < 0.001; 10 g vs. 50 g, *p* < 0.001; 25 g vs. 50 g, *p* < 0.001; C_max_: 10 g vs. 25 g, *p* < 0.001; 10 g vs. 50 g, *p* < 0.001; 25 g vs. 50 g, *p* = 0.001, see Table 1).

The absorption of erythritol was significantly slower with the 50 g load compared to the lower doses (absorption half-life t_ka,1/2_: 10 g vs. 50 g, *p* = 0.004, 25 g vs. 50 g, *p* = 0.002, see Table 1), suggesting a saturable process. Neither the elimination rate constant k_10_ of erythritol and its half-life t_k10,1/2_ nor the volume of erythritol distribution (V1) were significantly different between the treatment doses (Table 1).

**Figure 2:**
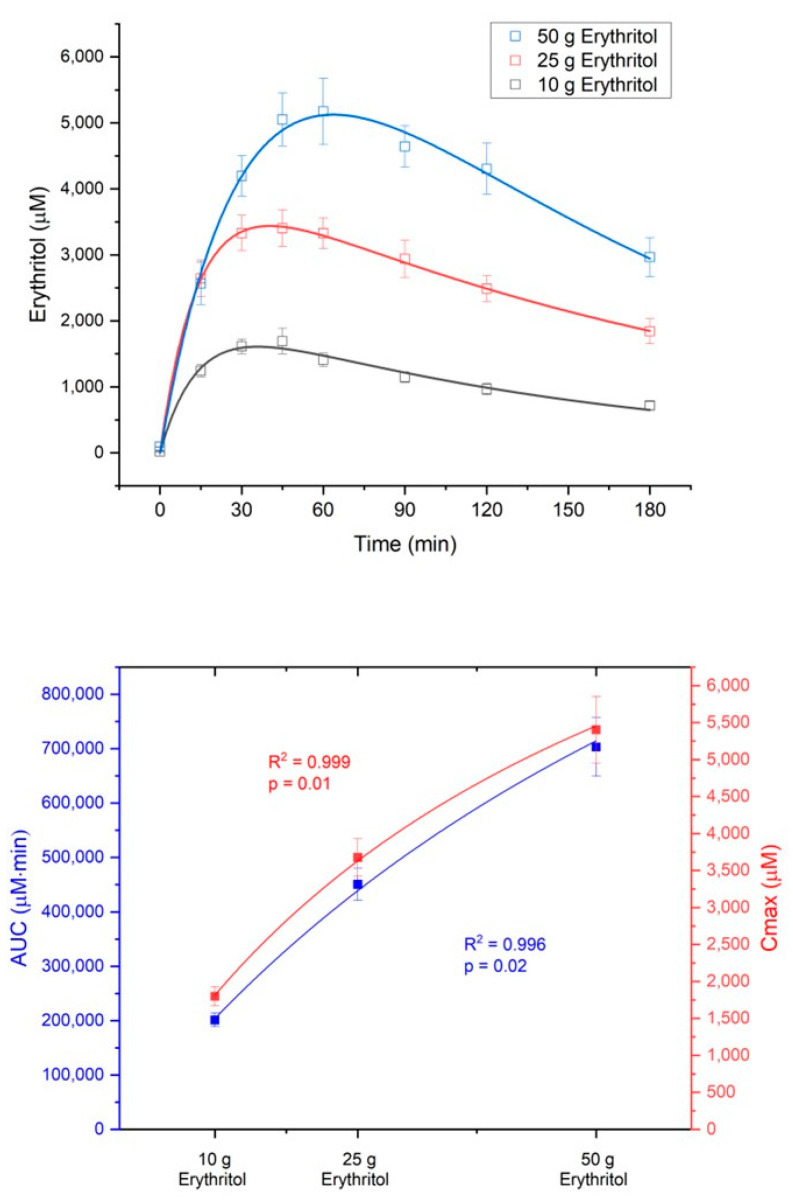
Dose-dependent absorption of erythritol. Upper part: concentration–time curves after administration of the three loads; lower part: dose-response for the area under the curve from 0 to 180 min (AUC180) and the maximum deviations from baseline (Cmax). Data are expressed as mean ± SEM. Data were best fit with a non-linear dose-response model.

Figure 2 shows the concentration-time curves and the dose-response diagram for AUC_180_ (dose-response: R^2^ = 0.996, *p* = 0.02) and C_max_ (dose-response: R^2^ = 0.999, *p* = 0.01) of erythritol after administration of the three loads.

Xylitol absorption was low and could not be detected in any of the participants after the 7 g dose, only in some of the participants after the 17 g dose and in all participants after the 35 g dose (data not shown).

### 2.2. Metabolization of Erythritol and Xylitol into Erythronate

**Figure 3:**
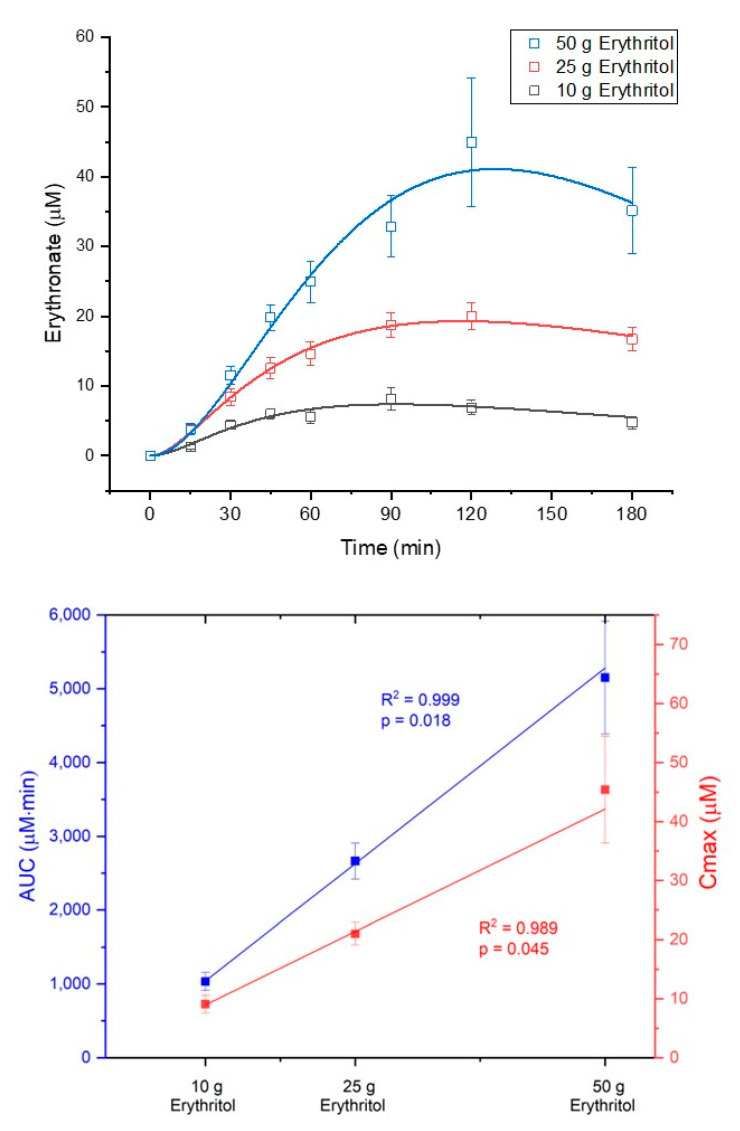
Dose-dependent metabolization of erythritol into erythronate. Upper part: concentration–time curves after administration of the three erythritol loads; lower part: dose–response for area under the curve from 0 to 180 min (AUC180) and maximum deviations from baseline (Cmax). Data are expressed as mean ± SEM.

The metabolization of erythritol into erythronate occurred in a dose-dependent manner. The AUC_180_ and C_max_ for erythronate plasma concentrations increased in response to the three intragastric loads of erythritol (AUC_180_: 10 g vs. 25 g, *p* = 0.069; 10 g vs. 50 g, *p* = 0.001; 25 g vs. 50 g, *p* = 0.002; C_max_: 10 g vs. 25 g, n.s.; 10 g vs. 50 g, *p* < 0.001; 25 g vs. 50 g, *p* = 0.01, see Table 2). Figure 3 shows the concentration–time curves of erythronate and the dose–response diagram for AUC_180_ (dose–response: R^2^ = 0.999, *p* = 0.018) and C_max_ (dose–response: R^2^ = 0.989, *p* = 0.045) of erythronate after administration of the three loads of erythritol. Neither the formation rate (k_12_), nor the elimination rate k_20_ of erythronate and its half-life t_k20,1/2_, nor the volume of erythronate distribution (V2) were significantly different between the treatment doses (Table 2).

**Figure 4:**
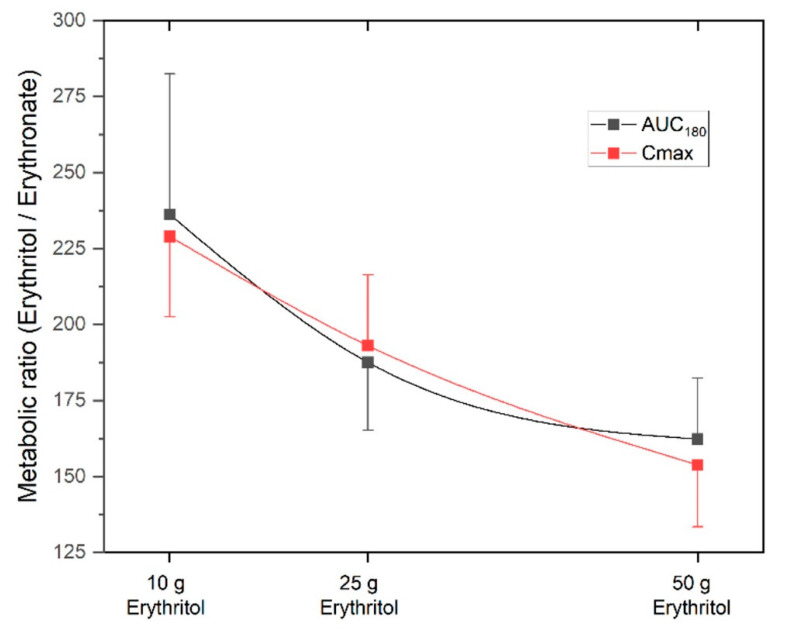
Metabolic ratios erythritol/erythronate for the three doses of erythritol. AUC_180_: area under the curve from 0 to 180 min, C_max_: maximum deviation from baseline. Data are expressed as mean ± SEM.

The metabolic ratio AUC_erythritol_/AUC_erythronate_ was highest with the 10 g erythritol load and decreased with higher doses (ratio AUC_180_ for 10 g: 236.2 ± 46.3 vs. 25 g: 187.6 ± 22.4 vs. 50 g: 162.3 ± 20.1, differences not significant). The C_max, erythritol_/C_max, erythronate_ ratio was also highest with the 10 g erythritol load compared to the higher doses (ratio C_max_ for 10 g: 229.0 ± 26.5 vs. 25 g: 193.1 ± 23.3 vs. 50 g: 153.8 ± 20.4, differences not significant). These decreasing metabolic ratios with higher doses of erythritol indicate that an increasing fraction of erythritol is metabolized into erythronate. Figure 4 shows the metabolic ratios depending on the different doses of erythritol. This phenomenon is also reflected by the non-linear dose-response for AUC_180_ and C_max_, as shown in the lower part of Figure 2.

The concentrations of erythronate in response to the intragastric loads of xylitol were under the detection limit of the analytical assay, indicating no metabolization of xylitol into erythronate at the doses applied in this study.

## 3. Discussion

This study aimed to determine the absorption of different intragastric doses of erythritol and xylitol and their potential metabolization into erythronate in healthy volunteers. The results show that: (i) the absorption of erythritol is dose-dependent and saturable; (ii) the absorption of xylitol is low; (iii) erythritol is metabolized into erythronate, the metabolization is dose-dependent and higher with high doses of erythritol; and (iv) there is no metabolization of xylitol into erythronate. The implications for human health remain to be determined.

The absorption results for erythritol are in line with other human studies showing that erythritol is rapidly absorbed [8,9,10]. However, the extent can only be estimated in comparison to an intravenous control. The dose-dependent absorption of erythritol found here confirms the results of Bornet et al. [8], who showed increasing plasma erythritol concentrations as a function of ingested doses (0.4 or 0.8 g/kg body weight). More importantly, we observed that the absorption of erythritol was slower with the highest dose (50 g) suggesting a saturable process. The slower absorption might explain gastrointestinal symptoms such as nausea, borborygmi, bloating, and diarrhea observed at high doses [5,20]. The hypothesis of a saturable absorption of erythritol at high doses is compatible with these observations.

Xylitol, on the other hand, was poorly absorbed in the present study. This contrasts with results showing absorption of at least 50% in healthy subjects [14]. In contrast, the previous study used a test solution consisting of xylitol with an equal amount of glucose, which is different from the current design. The addition of glucose might have affected the absorption of xylitol. Moreover, the authors estimated absorption by aspiration and analysis of ileal content (i.e., disappearance); of note, they did not find any xylitol in plasma samples one and two hours after ingestion [14].

**Figure 5:**
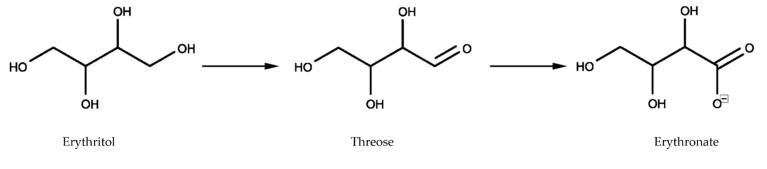
Oxidation reactions of erythritol into threose and erythronate.

Erythritol is metabolized into erythronate, confirming the findings of Hootman et al. [13]. In addition, we extend these findings by showing that this metabolization is dose-dependent and increases with high doses of erythritol. The metabolization process occurred, however, in minimal amounts: less than 1% of erythritol was converted into erythronate—which is less than Hootman et al. who reported a conversion rate of 5–10%. In their study, only three men were included and they received 50 g of oral erythritol 43 min after having consumed 2 g of labeled glucose. Although the dose is similar, the limited sample size, the route, and the timing of administration are important differences in the study design. Both studies agree that only on a small amount of erythritol is metabolized (<10%). We think that erythritol is converted by an alcohol dehydrogenase to threose, which is in turn probably further biochemically oxidized to erythronate (Figure 5).

The formation rate of erythronate did not significantly differ between the different doses of erythritol, although numerically, the metabolic ratio decreased with higher doses indicating that the metabolization increased. It is interesting to note that erythronate is eliminated faster than erythritol. The elimination rate of erythronate is about 0.02 min^−1^, while the elimination rate of erythritol is only 0.008 min^−1^ for the two lower doses and even lower (0.002 min^−1^) for the 50 g dose of erythritol. The reasons for these differences are unclear: the higher polarity of erythronate compared to erythritol could be an explanation, although both substances are quite hydrophilic. It is not known if erythronate is further metabolized or directly excreted in the urine.

We did not detect any metabolization of xylitol into erythronate. The metabolization of erythritol into erythronate might follow an oxidation reaction including the sugar erythrose. Therefore, xylitol has to be metabolized into erythrose before being further oxidized into erythronate. This reaction is possible requiring multiple steps: xylitol can be transformed into xylulose and then into xylulose-5-phosphate through enzymatic reactions. Xylulose-5-phosphate is an entry point into the pentose phosphate cycle [21]. Within this cycle, xylulose-5-phosphate can be metabolized into erythrose-4-phosphate, which, in turn, can be transformed into erythrose and further oxidized into erythronate. This process involves several steps; the resulting concentrations were not detectable within the dose range investigated here. Therefore, we conclude that xylitol is not metabolized into erythronate at the concentrations administered in this study.

Little is known about the role of erythronate in the human body. It seems that erythronate is an oxidative stress product [22,23]. It has been shown in feces of colorectal cancer patients, that erythronate correlated with the presence of Enterobacteriaceae, a potentially pathogenic group of bacteria [24]. In patients with liver cirrhosis, erythronate was associated with the severity of hepatorenal dysfunction and it was a significant predictor of mortality [25,26]. A stepwise increase in erythronate plasma concentration in patients with renal disease was observed with decreasing renal function [27]. In addition, erythronate was associated with the estimated glomerular filtration rate in the general European population, and the authors suggest that it may be an early marker of reduced kidney function [28]. However, before using erythronate as a marker of different conditions, more research is necessary and it must be taken into account that increasing amounts of erythritol is consumed, and that this consumption influences the circulating levels of erythronate.

Hootman et al. [13] found a positive association between circulating levels of erythritol and the incidence of central adiposity gain in nonobese young adults studied over nine months. They also showed that glucose can be metabolized into erythritol, and erythritol can be metabolized into erythronate. We and others hypothesize that the presence of erythritol and erythronate in the plasma of subjects who are not regularly consuming erythritol might serve as a marker of elevated blood sugar levels and oxidative stress, which are associated with central adiposity [29]. More research is needed to validate the role of erythronate metabolized from erythritol ingestion in the human body.

The present study has some limitations: first, it is an a posteriori analysis, and therefore, no pre-study sample size calculation was made. However, as the results of the main trial (dose-response effect on satiation hormones secretion) were significant [6,7], we can assume that the sample size was robust to detect a dose-dependent effect in the absorption of erythritol and xylitol and their conversion into erythronate. Second, the doses chosen might have been too low to observe the conversion of xylitol into erythronate. However, as mentioned before, the doses in this study were chosen to represent real life conditions and to limit gastrointestinal symptoms. Finally, we only assessed erythritol, xylitol, and erythronate concentrations in the plasma. It would be interesting to measure their concentration in urine and feces, too, to understand whether erythronate is further metabolized or directly eliminated.

In conclusion, erythritol is absorbed in a dose-dependent and saturable manner. It is metabolized in a small amount into erythronate, and this process is dose-dependent. The absorption of xylitol is low, and no metabolization into erythronate takes place at the doses used in this study. The implications for human health remain to be determined.

## 4. Materials and Methods

### 4.1. Study Approval

The trial was approved by the local ethical committee of Basel, Switzerland (Ethikkommission Nordwest- und Zentralschweiz; EKNZ 2016-01928) and was performed in compliance with the current version of the Declaration of Helsinki, the ICH-GCP, and national legal and regulatory requirements. Each participant gave written informed consent for the trial. The trial was registered at ClinicalTrial.gov under NCT03039478.

### 4.2. Participants

A total of 17 healthy normal-weight participants took part in the trial. The participants’ baseline characteristics are shown in Table 3. Participants were excluded if they suffered from acute infections, chronic diseases, or diseases of the gastrointestinal tract, if they took medications regularly, if they were pregnant, or if they consumed substances in abuse. In addition, none of the participants had a history of food allergies, dietary restrictions, or pre-existing consumption of erythritol or xylitol on a regular basis.

### 4.3. Study Design

This acute study was conducted as a parallel trial. The first twelve included participants were given erythritol; the following twelve participants were included in the xylitol arm. Within the arms, the doses were given in a randomized order. The trial was conducted double blind, meaning that the study participant, the person carrying out all tests, and the personnel performing the analyses of blood samples were blinded concerning the dosage assigned to the participant. Some participants (n = 7) participated in the xylitol arm after enrolling in the erythritol arm. That is why the total number of participants was only 17. For participants included in both arms, a wash-out phase of at least four days between the two interventions was respected.

### 4.4. Experimental Procedure

Participants were admitted to St. Clara Research Ltd. in the morning after a 10 h overnight fast. A feeding tube was placed to administer the substances intragastrically. This route of administration was chosen to bypass exteroceptive cues (e.g., taste and smell) and their associated hedonic responses and cognitions that may influence subjective ratings or even physiological/endocrine responses [30]. An antecubital catheter was inserted into a forearm vein for blood sampling. After taking fasting blood samples, participants received one of the following solutions (*t* = 0 min) directly into the stomach, over two minutes, in a randomized order, depending on the intervention arm:

For the erythritol arm:

- 10 g of erythritol dissolved in 300 mL tap water
- 25 g of erythritol dissolved in 300 mL tap water
- 50 g of erythritol dissolved in 300 mL tap water

For the xylitol arm:

- 7 g of xylitol dissolved in 300 mL tap water
- 17 g of xylitol dissolved in 300 mL tap water
- 35 g of xylitol dissolved in 300 mL tap water

The doses of erythritol were chosen to represent everyday life conditions. For example, 50 g erythritol dissolved in 300 mL corresponds to 30 g sucrose in 330 mL, the concentration found in common sweet beverages. Moreover, the gastrointestinal tolerance of 50 g of erythritol seems acceptable, as this dose only causes nausea and borborygmi, while 50 g of xylitol can cause bloating, colic, and watery feces in some subjects. Lower doses of erythritol do not cause any symptoms [20]. The doses of xylitol were chosen to be equisweet to erythritol.

**Figure 6:**
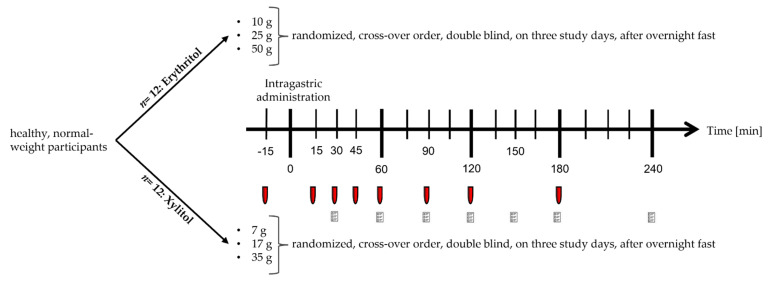
Diagram of the experimental procedure. The red tubes stand for blood sampling, the paper forms stand for gastrointestinal symptoms questionnaires.

After administration of the test solutions, blood samples were taken at regular time intervals (*t* = 15, 30, 45, 60, 90, 120, and 180 min). Participants were asked to rate GI symptoms at 30, 60, 90, 120, 150, 180, and 240 min after administration of the test solutions. The experimental procedure is depicted in Figure 6.

### 4.5. Blood Sample Collection and Processing

Blood samples were collected on ice into tubes containing EDTA (6 µmol/L blood) and a protease-inhibitor cocktail (Complete, EDTA-free, one tablet/50 mL blood, Roche, Mannheim, Germany). After centrifugation (4 °C at 3000 rpm for 10 min), plasma samples were processed into aliquots. The samples were then stored at −80 °C until analysis.

### 4.6. Materials

Erythritol and xylitol were purchased from Mithana GmbH (Zimmerwald, Switzerland).

### 4.7. Assessments of Erythritol, Xylitol, and Erythronate Concentrations

To analyze erythritol, xylitol, and erythronate, the plasma samples were first extracted with a solution of water/methanol (1/8) *v/v* containing the internal standard and dried at 55 °C on a vacuum centrifuge for one hour. The dried sample spots were then reconstituted in pyridine containing methoxyamine and derivated at 70 °C for 30 min. Before analysis, the samples were derivated a second time by adding N-Methyl-N-(trimethylsilyl)-trifluoracetamid at 40 °C for another 30 min. Finally, the concentration of erythritol, xylitol, and erythronate was assessed using gas chromatography-mass spectrometry with helium as carrier gas. In samples from the erythritol arm, xylitol was used as an internal standard; in samples from the xylitol arm, erythritol was used as the internal standard.

### 4.8. Statistical Analysis

This study is an a posteriori sample analysis. Therefore, no sample size calculation was made, and 12 participants per group was chosen for comparability and practicability. However, as the results of the main trial (dose-response of satiation hormones secretion) were significant [6,7], we can assume that the sample size is enough to detect a dose-dependent effect in the absorption of erythritol and xylitol and their conversion into erythronate.

**Figure 7:**
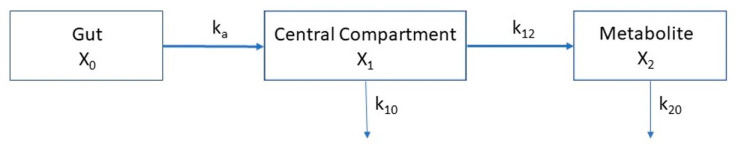
Three-compartment model for the absorption of erythritol/xylitol and the conversion into erythronate.

Molar concentrations of erythritol, xylitol, and erythronate were analyzed kinetically using the following system of coupled differential Equation (1), based on the three-compartment model depicted in Figure 7.

dX_0_/dt = −k_a_ ⋅ X_0_ dX_1_/dt = k_a_ ⋅ X_0_ − k_10_ ⋅ X_1_ − k_12_ ⋅ X_1_ dX_2_/dt = k_12_ ⋅ X_1_ − k_20_ ⋅ X_2_ (1)

This linear three-compartment model describes the mass transfer between the first compartment (X_0_, gut), from which the absorbable fraction of the erythritol/xylitol dose (F × dose, where F = bioavailability) is absorbed into the central compartment (X_1_, blood) by a linear process with a rate constant k_a_. Erythritol/xylitol in compartment X_1_ is either eliminated from the compartment (elimination rate constant k_10_) or metabolized into erythronate in the compartment X_2_. Although the formation of erythronate from erythritol/xylitol is done by enzymatic reaction, it could best be modeled by a linear process and was denoted by the formation rate constant k_12_. The elimination of erythronate from the metabolite compartment (X_2_, blood) is described by the elimination rate constant k_20_. The volumes of distribution of compartments X_1_ and X_2_, called V_1_ and V_2_, respectively, relate the masses to plasma concentration (y_1_ and y_2_), as shown in the following Equation (2):

y_1_(t) = X_1_(t)/V_1_ and y_2_(t) = X_2_(t)/V_2_. (2)

The initial conditions at t = 0 were set to X_0_(0) = molar doses, X_1_(0) = 0, and X_2_(0) = 0.

Data were modeled using Python programming language (version 3.8.5), and the Limfit module version 1.0.2 (Newville, M. et al., LMFIT: Non-Linear Least-Square Minimization and Curve-Fitting for Python (2021), https://zenodo.org/record/11813#.Yex0ZerMJZc) to numerically solve the Equations (1) and (2) and fit them to the observed concentrations of xylitol, erythritol, and erythronate.

The half-life of the respective rate constant k for absorption and elimination was calculated as shown in the following Equation (3):

t_k,1/2_ = ln (2)/k, ln = natural logarithm. (3)

The concentrations of erythritol, xylitol, and erythronate were baseline-corrected before analysis.

All statistical analysis was done using the statistical software package IBM SPSS Statistics for Windows, Version 27.0 (Armonk, NY, USA: IBM Corp.). Values were reported and displayed as means ± standard error of the mean (SEM) if not otherwise specified.

Data were compared between doses by linear mixed model analysis with Šidak correction for multiplicity of testing. Differences were considered to be statistically significant when *p* < 0.05.

## Figures and Tables

**Table 1:** Absorption of erythritol.

	A: 10 g Erythritol (n = 11)	B: 25 g Erythritol (n = 12)	C: 50 g Erythritol (n = 12)	*p-*Values
**AUC_180_ (mM·min)**	201.0 ± 12.7	450.6 ± 29.3	707.1 ± 53.9	A vs. B: *p* < 0.001 A vs. C: *p* < 0.001 B vs. C: *p <* 0.001
**C_max_ (µM)**	1810.6 ± 124.6	3676.9 ± 251.2	5404.3 ± 450.6	A vs. B: *p* < 0.001 A vs. C: *p <* 0.001 B vs. C: *p =* 0.001
**Absorption rate k_a_ (min^−1^)**	0.126 ± 0.183	0.374 ± 0.257	0.036 ± 0.031	All n.s.
**Absorption half-life t_ka,1/2_ (min)**	5.40 ± 1.16	4.88 ± 0.86	14.23 ± 2.66	A vs. B: n.s. A vs. C: *p* = 0.004 B vs. C: *p* = 0.002
**Elimination rate k10 (min^−1^)**	0.008 ± 0.002	0.008 ± 0.001	0.002 ± 0.001	All n.s.
**Elimination half-life t_ka,1/2_ (min)**	46.09 ± 5.68	51.41 ± 3.62	42.69 ± 5.24	All n.s.
**Volume of distribution V1 (L)**	38.50 ± 4.27	37.74 ± 2.43	50.95 ± 5.62	All n.s.

Data are expressed as mean ± SEM and reported from baseline. Linear mixed effect model analysis with Šidak correction for multiple testing. AUC_180_: area under the curve from 0 to 180 min, C_max_: maximum plasma concentration, n.s.: not significant.

**Table 2:** Metabolization of erythritol into erythronate.

	A: 10 g Erythritol (n = 11)	B: 25 g Erythritol (n = 12)	C: 50 g Erythritol (n = 12)	*p-*Values
**AUC_180_ erythronate (µM·min)**	1034.4 ± 122.8	2664.8 ± 241.6	5151.9 ± 763.2	A vs. B: *p =* 0.069 A vs. C: *p* = 0.001 B vs. C: *p =* 0.002
**C_max_ erythronate (µM)**	9.1 ± 1.5	21.0 ± 1.9	45.4 ± 9.1	A vs. B: n.s. A vs. C: *p <* 0.001 B vs. C: *p =* 0.01
**Formation rate k_12_ (min^−1^)**	0.0003 ± 0.00009	0.0002 ± 0.00030	0.0002 ± 0.00002	All n.s.
**Elimination rate k_20_ (min^−1-^)**	0.0229 ± 0.0019	0.0188 ± 0.0012	0.0210 ± 0.0018	All n.s.
**Elimination half-life t_k20,1/2_ (min)**	14.34 ± 1.54	16.89 ± 1.23	15.59 ± 1.46	All n.s.
**Volume of distribution V2 (L)**	54.39 ± 14.61	49.33 ± 7.69	39.21 ± 5.34	All n.s.

Data are expressed as mean ± SEM and reported from baseline. Linear mixed effect model analysis with Šidak correction for multiple testing. AUC180: area under the curve from 0 to 180 min, Cmax: maximum plasma concentration, n.s.: not significant.

**Table 3:** Participants’ baseline characteristics (mean ± SD (range)).

Parameter	Xylitol Group	Erythritol Group	*p-*Values
**Gender**	n = 12 (7♀, 5♂)	n = 12 (5♀, 7♂)	0.683 ^†^
**Age (yrs)**	25.6 ± 5.1 (23; 41)	26.2 ± 6.6 (18; 40)	0.810 ^§^
**Weight (kg)**	64.5 ± 9.5 (51.0; 82.9)	66.4 ± 8.3 (54.7; 82.9)	0.607 ^§^
**Height (m)**	1.74 ± 0.11 (1.61; 1.90)	1.75 ± 0.09 (1.65; 1.90)	0.836 ^§^
**BMI (kg/m^2^)**	21.2 ± 1.3 (19.4; 23.0)	21.7 ± 1.4 (19.4; 24.0)	0.422 ^§^

^†^ Chi-square test, ^§^ Analysis of variance, ♀ stands for women, ♂ stands for men, BMI: body mass index, SD: standard deviation.

## Data Availability

The data presented in this study are available on request from the corresponding authors.
